# The effects of eccentric phase tempo in squats on hypertrophy, strength, and contractile properties of the quadriceps femoris muscle

**DOI:** 10.3389/fphys.2024.1531926

**Published:** 2025-01-09

**Authors:** Filip Kojic, Danimir Mandic, Sasa Duric

**Affiliations:** ^1^ Faculty of Education, University of Belgrade, Belgrade, Serbia; ^2^ Liberal Arts Department, American University of the Middle East, Egaila, Kuwait

**Keywords:** tensiomiography (TMG), contraction time, radial displacement, vastus lateralis, cross-cectional area (CSA), one-repetition maximum (1RM)

## Abstract

**Introduction:**

The aim of the present study was to investigate the effects of eccentric phase tempo in squats on hypertrophy, strength, and contractile properties of the quadriceps femoris (QF) muscle.

**Methods:**

Eighteen participants (10 males and 8 females, age 24.0 ± 1.7 years) with no resistance training (RT) experience in the last 8 months were randomized into two groups, each following a 7 week squat resistance training (RT) protocol with either a fast eccentric (FE, 1 s eccentric/0 s isometric/1 s concentric/0 s isometric) or slow eccentric (SE, 4 s eccentric/0 s isometric/1 s concentric/0 s isometric) tempo. The training intensity (60%–70% RM), the number of sets (3–4), and the rest intervals (120 s) were consistent in both groups. The study measured changes in quadriceps cross-sectional area (CSA), one-repetition maximum (1RM) strength, and muscle contractile properties such as contraction time (Tc) and radial displacement (Dm), using tensiomyography (TMG). An ANCOVA model with baseline values as covariates was used to examine between-group differences.

**Results:**

Results showed significant strength gains in both groups, with the SE group achieving greater 1RM increases (effect size [ES] = 1.60 vs 0.99, p < 0.05). CSA increased for all QF muscles; however, the SE group exhibited significantly higher hypertrophy in the vastus lateralis (ES = 1.74 vs. 1.37, p < 0.05). TMG analysis revealed decreased Dm in the rectus femoris for both groups (p < 0.05), while Tc significantly (ES = 1.33, p < 0.01) increased in the SE group.

**Discussion:**

These findings suggest that slower eccentric tempo in RT may optimize vastus lateralis hypertrophy and enhance strength while promoting muscle fiber-type specificity, contributing to the understanding of eccentric training’s role in muscle adaptation.

## Introduction

Modifying resistance training (RT) variables like intensity, volume, rest periods, contraction duration, and range of motion can significantly influence muscle adaptations (Iversen et al., 2021; Azevedo et al., 2022; Diniz et al., 2022). An often overlooked variable is contraction duration or tempo, which has recently gained attention for its effects on muscle hypertrophy and strength improvements (Schoenfeld et al., 2015; Wilk et al., 2020). Tempo, defined as the duration of the eccentric, isometric, and concentric phases of a movement, directly influences time under tension (TUT) and impacts other RT variables such as volume and intensity (Burd et al., 2012; Wilk et al., 2021; Suchomel et al., 2019). Repetition tempo is typically expressed as a four-digit sequence (e.g., 3/0/2/0), with each digit indicating the duration (in seconds) of the eccentric, isometric and concentric phases (Wilk et al., 2020; Wilk et al., 2021). Based on the literature to date, extremely slow repetitions (over 10 s per repetition) appear to be inferior for hypertrophy, whereas repetition durations between 0.5 and 8 s are likely to be more effective for muscle growth (Schoenfeld et al., 2015). In terms of strength development, both quick and moderately slow tempos appear to yield similar results (Davies et al., 2016). However, most previous studies have either used the equal duration of eccentric and concentric phases or manipulated only the duration of the concentric phase, and there is limited data showing how the duration of the eccentric phase affects muscular adaptations (Schoenfeld et al., 2015; Davies et al., 2016).

The rationale that prolonged eccentric contractions enhance muscle adaptations stems from the muscle’s capacity to resist 20%–60% more force during the eccentric phase than during the concentric phase (Ratamess et al., 2009). This has led to the hypothesis that either increasing the eccentric load or extending the duration of the eccentric phase can promote greater muscle growth and strength (Wagle et al., 2017). Some research supports this, as slower eccentric contractions (lasting 3–6 s) have been associated with increased metabolic stress and muscle damage, both key mechanisms for muscle growth (Wilk et al., 2020), although the role of muscle damage in hypertrophy has recently been questioned (Iversen et al., 2021). In this context, recent review papers by Wilk et al. (2021) and Moreno-Villanueva et al. (2022) suggest that a tempo configuration combining faster concentric and slower eccentric contractions (e.g., 4/0/1/0) may optimize strength gains, although data in this area are still limited. Conversely, longer concentric phases, while increasing TUT, appear to be less effective for strength development, possibly due to a reduction in neural adaptations associated with slower concentric training (Pareja-Blanco et al., 2014; Davies et al., 2016). This relates specifically to motor unit recruitment, firing frequency and synchronization, as slower concentric movements may fail to sufficiently stimulate these neural pathways because they decrease the demand for rapid force production and explosive activation, both of which are critical for maximizing strength (Pareja-Blanco et al., 2014). Interestingly, there is speculation that lower-body muscles, particularly the quadriceps femoris (QF), may respond better to prolonged contraction durations compared to upper-body muscles such as the biceps brachii, although the evidence for this is still limited (Hackett et al., 2018). This idea is based on the concept that the QF, as a large and multi-joint muscle group, has a diverse fiber composition, with a greater proportion of slow-twich fibers that may respond more favorably to increased TUT and prolonged contraction durations (Hackett et al., 2018; Azevedo et al., 2022). Such properties make QF an intriguing target for exploring the specific effects of tempo manipulation in RT.

The effects of varying eccentric tempos on lower-body muscles, particularly on strength and hypertrophy, have only been researched to a limited extent. Studies that have focused on the QF have generally reported similar hypertrophy outcomes regardless of tempo (Mike et al., 2017; Shibata et al., 2021; Pearson et al., 2022). However, these studies tend to assess the QF as a whole potentially overlooking uneven growth of its individual muscles, namely, the vastus medialis, vastus lateralis and rectus femoris. This distinction is important, as QF muscles often display inhomogeneous growth patterns, particularly following exercises such as the squat and knee extension (Zabaleta-Korta et al., 2020). Two studies (Azevedo et al., 2022; Diniz et al., 2022) have examined whether variation in tempo during knee-extension RT influences uneven hypertrophy of the QF. In a study by Azevedo et al. (2022), participants performed unilateral knee extension RT for 8 weeks with one leg assigned to a fast concentric/slow eccentric protocol (4/0/1/0) and the other assigned to a fast concentric/fast eccentric protocol (2/0/1/0). Both protocols resulted in hypertrophy of the QF, but the vastus medialis showed greater growth with prolonged eccentric contractions. Another study found that prolonged eccentric and shortened concentric contractions (5/0/1/0) resulted in greater hypertrophy in both the vastus medialis and vastus lateralis compared to a protocol in which the eccentric and concentric phases each lasted 3 s (3/0/3/0) (Diniz et al., 2022). Based on these findings, it appears that alternating the duration of the eccentric contraction may result in region-specific hypertrophy within the QF muscles. Interestingly, no study to date has investigated how different durations of contraction during squat-based RT affect the QF intermuscular hypertrophy.

Beyond hypertrophy and strength gains, assessment of involuntary muscle contractile properties can provide additional insights into how different RT protocols influence muscular adaptations (Macgregor et al., 2018). Tensiomyography (TMG) has emerged as a valuable tool in this regard, particularly for measuring radial displacement (Dm) and contraction time (Tc) (Langen et al., 2022). Specifically, TMG Dm assesses the spatial displacement of the muscle, providing a measure of muscle stiffness or tone (lower Dm values indicate higher stiffness and *vice versa*). Several studies have shown that chronic changes in Dm may predict muscle hypertrophy (Wilson et al., 2019; Šimunič et al., 2019; Kojić et al., 2022). On the other hand, TMG Tc has been associated with muscle fiber type, with lower Tc values being associated with a higher proportion of slow-twitch fibers (Dahmane et al., 2005; Simunic et al., 2011). Moreover, Simunic et al. (2011) developed a regression formula to estimate the composition of slow-twitch muscle fibers in the vastus lateralis based on Tc values, demonstrating the usefulness of TMG in assessing fiber composition in lower-body muscles.

Considering this and the fact that limited research has been conducted on the effects of eccentric tempo RT on lower-body muscles, further investigation is needed to better understand its effects. Accortingly, the present study was designed to comprehensively investigate how variation in eccentric phase tempo during squats influences intermuscular hypertrophy, strength gains and TMG adaptations in the QF muscles. Specifically, we examined individual QF muscles (the vastii and rectus femoris) to determine whether manipulating contraction duration leads to region-specific hypertrophy. In addition, we used TMG to gain deeper insights into the neuromuscular adaptations resulting from the different eccentric phase durations. Based on previous studies, we hypothesized that different contraction durations would result in uneven growth within the QF, while strength gains would remain similar in both protocols.

## Methods

### Participants

The participants (n = 18, 10 males and eight females, age 24.0 ± 1.7 years, height 1.75 ± 0.07 m, weight 69.50 ± 10.48 kg) were healthy individuals who had not engaged in RT activities in the past 8 months. None of them were professional athletes and had no chronic musculoskeletal diseases or injuries that could affect the results of the study. Participants were fully informed about the experimental procedures and potential risks and gave written informed consent before joining the study. They were instructed to maintain their usual diet throughout the experiment, avoid dietary supplements, and refrain from any physical activity for at least 48 h prior to testing. As part of their normal academic curriculum, participants engaged in six to 8 h of low-to high-intensity exercise per week. They had no chronic diseases, cardiac issues or recent musculoskeletal injuries and were informed of the potential risks associated with the testing protocol. The participants were blinded to the research question. The study was approved by the Institutional Ethics Committee (protocol code: 2316/19–2) and was conducted in accordance with the Declaration of Helsinki. It was registered at ClinicalTrials.gov (accessed on 14 April 2021) under the identifier NCT04845295.

### Experimental design

The aim of the study was to compare the effects of two different squat RT programs on hypertrophy, strength gains and the contractile properties of the knee extensors. Participants were randomly assigne, using a computer-generated randomization protocol, to one of two groups: the 1 s eccentric/0 s isometric/1 s concentric/0 s isometric group (FE) or the 4 s eccentric/0 s isometric/1 s concentric/0 s isometric group (SE). The key difference between these groups was the duration of the eccentric phase, with one group performing a 1 s eccentric phase and the other a 4 s eccentric phase, while the concentric phase was kept constant at 1 s. Both groups completed a 7 week squat RT program consisting of 14 training sessions (2 sessions per week). To prepare for the training intervention, the participants underwent a 2 week familiarization phase. This included a demonstration of the squat exercise, proper use of the equipment, and practice at the prescribed tempos for both groups. Participants performed submaximal repetitions during this sessions to adapt to the exercise and tempo. Measurements of four knee extensor cross-sectional area (CSA) and one-repetition maximum (1RM) were taken after the familiarization phase; 2 days before and after the training intervention. In addition, contractile properties were assessed 5 days before and after the experiment (Šimunič et al., 2019).

### Data acquisition

#### 1RM

Muscle strength was assessed using a 1RM test for the parallel barbell squat exercise before and after the intervention. The testing followed the protocol recommended by Baechle and Earle (2008): after a 10 min warm-up period, participants first completed 8–10 repetitions at approximately 50% of their estimated 1RM, followed by two to three repetitions at 60%–80% of their estimated 1RM. Each participant had up to five attempts to lift the maximum weight, with 3 min rest intervals between each attempt.

The 1RM test was performed with a straight barbell placed across the shoulders above the acromion with the feet shoulder-width apart. The range of motion included a complete concentric movement in which the body was brought into an upright position. During the eccentric phase, participants lowered the weight until their femurs were parallel to the floor, ensuring that the greater trochanter and lateral epicondyle of the femur were aligned at the same level (Earp et al., 2015). The individual depth of the squat was measured for each participant and an elastic band was positioned at the appropriate height. Participants were required to touch the band during the eccentric phase to confirm that they had achieved a 90-degree knee angle. The weights were selected by coach/researcher only.

#### CSA

The cross-sectional area (CSA) of the four quadriceps muscles—vastus medialis (VM), vastus lateralis (VL), vastus intermedius (VI), and rectus femoris (RF)—was assessed using a Siemens Antares ultrasound machine (Erlangen, Germany) equipped with a variable high-frequency transducer (ranging from 7 MHz to 13 MHz). The 2D ellipse diagnostic method was applied. Imaging was conducted after about 10 min rest in supine position on right leg in full extension. The ultrasound probe was transversally placed on the skin with minimal compression with conductive gel used for acoustic coupling on the skins surface. Brightness and depth were standardized across all participants, while the CSA was determined by ultrasound unit itself. The imaging technique used was previously validated by our research group (Kojić et al., 2021).

For the RF, the CSA was measured at three-fifths of the distance between the anterior superior iliac spine and the superior border of the patella. The CSA of the VI was measured at the midpoint between the anterior superior iliac spine and the proximal border of the patella. The VL CSA was measured at the midpoint of the femoral length, defined as 50% of the distance between the greater trochanter and the lateral condyle. The VM was measured at the distal portion just above the medial side of the patella.

An experienced and trained radiologist performed the image acquisition. Participants selected their dominant leg by answering the question: “Which leg would you use to kick a ball?” This leg was then used for the analysis. The same experienced researchers conducted both the pretest and posttest measurements.

#### TMG

The contractile properties of the RF and VL muscles were assessed using TMG according to the manufacturer’s instructions (TMG-BMC, Ljubljana, Slovenia). The analysis focused on two key parameters: contraction time (Tc) and radial displacement (Dm).

The testing was performed in the supine position, with the dominant leg supported at a knee angle of 120°. To ensure accurate sensor placement, participants were asked to perform a voluntary contraction so that the exact position of the TMG sensor could be determined by palpation. Two self-adhesive electrodes (Pals Platinum, model 895220 with multi-stick gel, Axelgaard Manufacturing Co. Ltd.) were then placed 3 cm proximal and distal to the marked site and delivered an electrical impulse. A sensor (GK40, Panoptik, Ljubljana, Slovenia) was positioned between the electrodes to detect muscle changes triggered by the electrical stimulation. The initial impulse was set to 25 mA and gradually increased by 10 mA until the muscle no longer responded to the stimulus. A 10 s pause between pulses allowed the muscle to fully relax. The two best results were recorded and the software calculated the mean value (Wilson et al., 2019; Simunic et al., 2011; Kojić et al., 2021). Both pretest and posttest TMG assessments were conducted in the morning by the same experienced specialist to ensure consistency.

### Experimental intervention

The treatment for both experimental groups consisted of barbell back squats, with a minimum interval of 48 h between two sessions, performed at approximately the same time of day. The differences were in the tempo of the exercise, particularly by manipulating the duration of the eccentric phase. The duration of the repetitions was controlled with a metronome. Researchers provided verbal cues and ensured that participants maintained the designated tempo throughout the sessions. The load and the number of sets were identical for both groups: in the first 3 weeks, the participants performed the exercise with a relative load of 60% RM for 3 sets, while in the last 4 weeks, the relative load was increased to 70% RM for 4 sets. All repetitions were performed until the participants could no longer execute the contraction properly (muscle failure) or could no longer maintain the prescribed tempo. The rest periods between sets were 2 min. We chose to have participants perform repetitions until muscular failure rather than prescribing an arbitrary number of repetitions, as this approach ensures a consistent stimulus for all individuals by tailoring the exercise to each participant’s volitional fatigue (Dankel et al., 2017). Each session was supervised by experienced researchers to ensure adherence to the protocol and proper execution of the exercises.

### Statistical analysis

Statistical analyses were performed using IBM SPSS Statistics software (Version 20, SPSS Inc., Chicago, IL, United States). The test-retest reliability of the CSA, 1RM and TMG measurements has already been reported (Kojić et al., 2021), with all diagnostic methods showing excellent reliability (ICC = 0.939–0.997, p < 0.01). Before conducting any analyses, the data were checked for normality and compliance with the relevant assumptions for each statistical test. The Shapiro-Wilk test was used to assess the normality of the data distribution. Baseline between-group differences were tested using an independent t-test, and homogeneity of variances was assessed.

Differences in the number of repetitions per set, training volume and TUT between the FE and SE groups were analyzed with independent t-tests. Within-group pre-to post-intervention changes in BB thickness, 1RM and TMG parameters were assessed using paired (dependent) t-tests. The effect size (ES) was calculated as Cohen’s d using G*Power software (Version 3.1, University of Kiel, Germany) based on the guidelines for untrained individuals proposed by Rhea et al. (2003). The ES thresholds were defined as follows: trivial (<0.50), small (0.50–1.25), moderate (1.25–1.90), and large (>2.0).

A one-way ANCOVA with baseline values as covariates was used to examine differences in the tested variables in the posttest between the FE and SE groups. If the ANCOVA indicated statistically significant results, the Bonferroni *post hoc* test was applied to further investigate the group differences. Effect sizes were also determined using G*Power software, with partial Eta squared values obtained from ANCOVA. All data are presented as mean ± SD, and statistical significance was set at p ≤ 0.05.

## Results

### Training volume and TUT

The FE group achieved a significantly higher training volume than the SE group (for 8.2 more reps, ES = 1.45, p < 0.01). In particular, the FE group completed significantly more repetitions across all three sets: 2.6 more repetitions in the first set (ES = 0.85, p < 0.05), 3.2 more in the second set (ES = 1.52, p < 0.01) and 2.4 more in the third set (ES = 1.28, p < 0.05). Conversely, the TUT was significantly and nearly twice higher in favor of the SE group (ES = 3.15, p < 0.01) (Table 1).

**TABLE 1:** Training volume and TUT in both experimental groups.

	FE group	SE group	*p*
Set 1 (reps)	14.5 ± 2.1*	11.9 ± 1.8	0.019
Set 2 (reps)	13.1 ± 2.6**	9.9 ± 1.4	0.009
Set 3 (reps)	11.1 ± 2.7*	8.7 ± 1.5	0.044
Volume (sum of reps in 3 sets)	38.7 ± 7.1**	30.5 ± 4.0	0.009
TUT (s)	77.5 ± 14.2	152.5 ± 20.0**	0.001

*p < 0.05; **p < 0.01.

### Quadriceps CSA, 1RM and TMG parameters

Both experimental groups significantly improved their 1RM compared to the pretest (FE group: increase of 5.6 kg, p < 0.05, ES = 0.99; SE group: increase of 11.1 kg, p < 0.01, ES = 1.60). It is noteworthy that the strength improvement in the SE group was significantly greater than in the FE group (F = 5.31, p < 0.05, ηp^2^ = 0.64) (Table 2).

**TABLE 2:** Changes in QF cross-sectional area (CSA), one-repetition maximum (1RM), and Tensiomyography (TMG) parameters for the FE and SE group before and after the intervention.

	FE group (4 females, 5 males)		SE group (4 females, 5 males)		p (ANCOVA)	ηp^2^ (ANCOVA)
	Pretest	Posttest	Pretest	Posttest		
CSA QF (cm^2^)	13.04 ± 3.42	13.62 ± 3.35**	12.06 ± 2.20	12.69 ± 2.24**	0.838	0.05
CSA RF (cm^2^)	3.23 ± 1.05	3.45 ± 0.92*	3.50 ± 0.91	3.67 ± 0.92*	0.846	0.05
CSA VI (cm^2^)	2.86 ± 1.18	3.04 ± 1.35*	2.60 ± 0.70	2.76 ± 0.70*	0.979	0.01
CSA VM (cm^2^)	3.21 ± 0.83	3.32 ± 0.82*	2.67 ± 0.49	2.80 ± 0.50**	0.821	0.06
CSA VL (cm^2^)	3.74 ± 0.83	3.81 ± 0.84**	3.28 ± 0.28	3.46 ± 0.33**	0.021*	0.69
1RM (kg)	101.87 ± 28.78	107.5 ± 31.96*	92.78 ± 19.22	103.89 ± 23.15**	0.041*	0.61
Dm RF (mm)	9.04 ± 2.49	7.40 ± 2.62*	10.93 ± 2.58	9.42 ± 2.46*	0.570	0.16
Tc RF (ms)	26.44 ± 3.54	25.05 ± 4.77	26.99 ± 3.04	30.52 ± 3.68**	0.012*	0.77
Dm VL (mm)	6.16 ± 2.41	5.21 ± 1.57	7.02 ± 1.92	6.58 ± 1.39	0.060	0.54
Tc VL (ms)	22.29 ± 4.24	21.61 ± 3.29	22.11 ± 2.36	24.30 ± 2.60**	0.031*	0.64

*p < 0.05; **p < 0.01; CSA, cross-sectional area; QF, quadriceps femoris; RF, rectus femoris; VI, vastus intermedius; VM, vastus medialis; VL, vastus lateralis; Dm, radial displacement; Tc, contraction time.

The training intervention led to a significant increase in the total CSA of QF in the posttest compared to the pretest (FE group: increase of 57.7 mm^2^, p < 0.01, ES = 1.66; SE group: increase of 63.7 mm^2^, p < 0.01, ES = 1.52), with no differences between the groups (F = 0.04, p = 0.838, ηp^2^ = 0.05). The CSA of RF increased significantly in both groups (FE group: increase of 21.4 mm^2^, p < 0.05, ES = 0.85; SE group: increase of 17.2 mm^2^, p < 0.05, ES = 1.02), with no differences between the groups (F = 0.04, p = 0.846, ηp^2^ = 0.05). Both groups also showed a significant increase in the CSA of VI compared to the pretest (FE group: increase of 17.6 mm^2^, p < 0.05, ES = 0.82; SE group: increase of 15.4 mm^2^, p < 0.05, ES = 1.00), with no differences between the groups (F = 0.001, p = 0.979, ηp^2^ = 0.007). Both FE and SE groups significantly increased the CSA of VM compared to the pretest (FE group: increase of 11.4 mm^2^, p < 0.05, ES = 1.16; SE group: increase of 12.8 mm^2^, p < 0.01, ES = 1.87), with no differences between the groups (F = 0.053, p = 0.821, ηp^2^ = 0.06). The VL CSA also increased significantly in both groups (FE group: increase of 7.4 mm^2^, p < 0.01, ES = 1.37; SE group: increase of 18.2 mm^2^, p < 0.01, ES = 1.74). However, the ANCOVA revealed a significantly greater increase in VL CSA in favor of SE group compared to FE group (F = 6.77, p = 0.021, ηp^2^ = 0.69) (Table 3).

In both the FE and SE groups, the Dm of the RF muscle decreased significantly compared to pretest (FE group: decrease of 1.6 mm, p < 0.05, ES = 1.07; SE group: decrease of 1.5 mm, p < 0.05, ES = 0.98), with no significant differences between the groups (F = 0.10, p = 0.758, ηp^2^ = 0.09). In addition, Tc significantly increased in the SE group (increase of 3.8 m, p < 0.01, ES = 2.03), while the decrease in Tc in the FE group did not reach statistical significance (decrease of 1.6 m, p = 0.355, VE = 0.35). The ANCOVA analysis revealed significant differences between the groups regarding changes in the Tc parameter compared to the pretest for the RF muscle: the FE group slightly reduced Tc, whereas the SE group significantly increased it (F = 8.82, p < 0.01, ηp^2^ = 0.82). For the VL muscle, the training intervention did not cause any significant changes in the Dm parameter (FE group: decrease of 0.9 mm, p = 0.076, ES = 0.74; SE group: decrease of 0.4 mm, p = 0.388, ES = 0.32). However, the Tc parameter significantly increased in the SE group (increase of 2.2 m, p < 0.05, ES = 1.33), while no significant changes were observed in the FE group (p = 0.620). The ANCOVA analysis showed significant differences between groups in the changes of the Tc parameter compared to the pretest for the VL muscle: the FE group slightly reduced Tc, whereas the SE group significantly increased it (F = 4.90, p < 0.05, ηp^2^ = 0.64) (Table 2).

## Discussion

The aim of this study was to investigate the effects of two squat RT modalities, differing in the duration of the eccentric phase, on the size, strength and contractile properties of the knee extensor muscles. Muscle hypertrophy was assessed across four QF muscles using ultrasound diagnostics. The main findings indicate that a prolonged eccentric tempo results in greater TUT but a reduced training volume. Despite this, the hypertrophic response of the QF muscles remained similar between tempos, except for the vastus lateralis (VL). The slower eccentric tempo (4/0/1/0) resulted in a significantly greater increase in CSA of the VL compared to the faster tempo (1/0/1/0). Lower-body muscle strength was assessed using a 1RM test for the parallel squat, and the results revealed that the slower eccentric tempo was superior to the faster tempo in increasing 1RM strength, but on the edge of statistical significance (p = 0.041). In addition, the contractile properties such as Dm and Tc parameters of the RF and VL muscles were assessed using TMG. Both training groups showed increased stiffness (evidenced by reduced Dm values) in the RF muscle, but not in the VL muscle. However, the novel finding of the study is that the slower eccentric tempo led to a significant increase in TMG Tc, a parameter related to the proportions of slow twich fibers. In contrast, the fast eccentric tempo decreased Tc values, although this did not reach statistical significance.

As anticipated, TUT was significantly greater following slower eccentric squats, supporting previous research highlighting the importance of repetition duration for TUT (Wilk et al., 2020; Wilk et al., 2021; Schoenfeld et al., 2015). In contrast, the FE group achieved a higher number of repetitions, resulting in a greater training volume (as the number of sets was identical for both groups). The rapid contractions during the eccentric phase facilitate the utilisation of elastic energy in the concentric phase (Douglas et al., 2017), which accounts for the increased repetitions and training volume observed in the FE group. Conversely, some studies have reported no significant differences in TUT (Shibata et al., 2021) or the number of repetitions (Wilk et al., 2018) across varying RT tempos. For example, Shibata et al. (2021) found that both a faster tempo (2/0/2/0) and a slower tempo (4/0/2/0) produced similar TUT outcomes. Similarly, Wilk et al. (2018) observed no difference in the total number of repetitions performed to failure between the 5/0/3/0 and 6/0/4/0 tempos. Notably, these studies used a 2 s difference per repetition between the tempos, whereas our study used a 3 s difference between the FE and SE conditions. Hence, it appears that a difference in repetition duration of 3 s or more is necessary to elicit significant changes in both TUT and number of repetitions, ultimately affecting training volume.

In terms of muscle hypertrophy, both training protocols resulted in a significant increase in total QF CSA, with no notable differences between the two groups, which is consistent with the findings from Shibata et al. (2021). Their study demonstrated that both slower (4/0/2/0) and faster (2/0/2/0) tempos during squats resulted in similar changes in overall QF CSA. However, their study measured total anterior thigh size without isolating individual muscles, making it unclear whether tempo variations affect hypertrophy in specific quadriceps muscles. In contrast, our study found that the slower eccentric tempo (4/0/1/0) resulted in significantly greater hypertrophy in the VL compared to the faster tempo (1/0/1/0), suggesting that manipulation of tempo in RT may induce intermuscular variability in hypertrophy. This observation is consistent with recent studies (Azevedo et al., 2022; Pearson et al., 2022) showing that varying contraction durations during knee-extension RT lead to region-specific hypertrophy within the QF. The greater growth of the VL in the slower eccentric tempo group may be explained by its higher level of activation during the eccentric phase of the squat, compared to the VM and RF muscles (Pincivero et al., 2006). Therefore, the increased TUT in the slower tempo group likely contributed to this greater hypertrophy in the VL. Moreover, given the dominant role of the VL in parallel squats (Pincivero et al., 2006), the greater VL hypertrophy in the SE group likely contributed to the greater increase in 1RM compared to the FE group. In contrast, neither (Mike et al. (2017) nor Shibata et al. (2021) observed superior strength gains following slower tempo eccentric squat RT. However, it should be noted that by Shibata et al. (2021) the groups performing different squat RT protocols were matched by TUT. In the study by Mike et al. (2017), although significance was not reached, a slightly higher increase in squat 1RM (13.2% vs 8.8%) was observed after longer eccentric durations (4/0/2/0) compared to shorter durations (2/0/2/0). In addition, the difference between FE and SE groups in the present study was significant, but on the edge of statistical significance (ES = 0.99 vs 1.60, p = 0.041).

Previous studies investigating the effects of RT on TMG parameters have primarily focused on acute effects. In fact, only one study investigated chronic changes in TMG following 8 weeks of squat RT and found a significant decrease in the Dm value for the RF and not the VL muscle (Wilson et al., 2019), which is consistent with the present results. The lack of significant change in the Dm value of the VL could be attributed to its postural role and pre-existing stiffness, as evidenced by the lower baseline Dm values compared to the RF. In fact, the training stimulus may not drastically alter the stiffness of the postural muscles in contrast to the non-postural muscles (Kojić et al., 2021). In addition, the present results showed that both training protocols induced similar changes in Dm for the RF and VL regardles of tempo. This is consistent with a previous study (Kojić et al., 2022) on upper-body muscles in which slower (4/0/1/0) and faster eccentric (1/0/1/0) tempos resulted in similar changes in Dm of the biceps brachii. These results suggest that RT-induced changes in muscle stiffness occur independently of TUT.

One of the interesting findings of the present study is that the TMG parameter Tc significantly increased only after slower eccentric RT, while it inversely decreased following faster eccentric tempo. Previously, TMG Tc has been found to be identified as a significant indicator of muscle composition, with higher values suggesting a predominance of type I fibers (Simunic et al., 2011; Dahmane et al., 2005). If this interpretation is correct, our study provides novel insights into how RT tempo affects muscle composition, suggesting that hypertrophy of VL and RF muscles at slower eccentric tempo is primarily due to hypertophy of slow-twitch fibers. Unfortunately, the effects of RT tempo on muscle fiber composition have only been studied to a limited extent, making direct comparison with previous studies difficult. To our knowledge, only one previous study (Gilles et al., 2006) has investigated the effects of RT tempo on muscle fiber composition and found that a slower eccentric tempo (6/0/2/0) increased VL size primarily through hypertrophy of slow-twitch fibers. This finding is consistent with our results. In addition, Hackett et al. (2018) proposed that the effectiveness of TUT on hypertrophy may depend on muscle fiber composition, with slower contractions being more effective for muscles rich in type I fibers. Since the VL has a relatively high proportion of slow-twitch fibers (Simunic et al., 2011), this could further explain why the slower tempo was more effective for inducing VL hypertrophy in our study.

## Strengths and limitations

The primary strength of this study lies in its comprehensive approach to investigate whether manipulating the tempo of squat RT affects physiological and contractile adaptations in the knee extensor muscles. In this study, we examined individual QF muscles to determine whether variations in contraction duration led to region-specific (intermuscular) hypertrophy. In addition, we used TMG to gain deeper insights into the neuromuscular adaptations associated with different eccentric phase durations. To the best of our knowledge, this is the first study to explore the effects of squat RT with different eccentric tempos on both intermuscular hypertrophy and contractile properties of the QF.

Despite the strengths of the present study, some limitations should be considered. First, the study did not include a control group, although the design of the present study did not require a control group because the FE group could be considered as such, as its tempo reflects the standard pace typically used during exercise. Second, we measured the CSA of the QF muscles at a single site. Given that skeletal muscles may exhibit intramuscular hypertrophy (i.e., proximal, central, or distal growth) in addition to intermuscular hypertrophy (Pearson et al., 2022), it is possible that greater growth occurred in unmeasured regions. Third, although participants were instructed to maintain their usual dietary and exercise habits, we did not systematically monitor these behaviors, which could have potentially influenced the results. The next limitation relates to the duration of the intervention: although a 7 week duration may provide valuable insights, it may not represent the optimal time frame for observing maximal adaptations. In addition, participants' menstrual cycles were not monitored, which may also be a limitation of this study. However, recent evidence suggests that ovulation does not have a significant effect on muscle size or strength adaptations in women, although this remains a potential confounding factor (Kuehne et al., 2021). Finally, while this study focused primarily on RT tempo, it is important to note that differences in TUT and training volume were observed between the experimental groups. These differences should be considered when interpreting the results, as they may have influenced the results.

## Conclusion

The results of this study showed that the tempo of the eccentric squat phase determines TUT and training volume and had a significant influence on hypertophy, strength and contractile properties of the QF muscles. Both the fast eccentric and slow eccentric tempos resulted in an overall increase in strength and CSA of the QF, with the SE group showing significantly greater improvements in 1RM strength and hypertrophy specifically in the VL. In addition, the TMG analysis showed that the slow eccentric tempo resulted in a significant increase in Tc, suggesting an adaptation in favor of hypertrophy of slow-twitch muscle fibers. The decrease in Dm in the RF for both groups emphasized increased muscle stiffness as a common adaptation, independent of the tempo. These results suggest that slower eccentric tempos may be more effective in targeting hypertrophy in specific QF muscles, such as the VL, and could support fiber-type specific training adaptations.

## Data Availability

The raw data supporting the conclusions of this article will be made available by the authors, without undue reservation.
